# Digital Gamification Tool (Let’s Control Flu) to Increase Vaccination Coverage Rates: Proposal for Algorithm Development

**DOI:** 10.2196/55613

**Published:** 2024-09-10

**Authors:** Henrique Lopes, Ricardo Baptista-Leite, Catarina Hermenegildo, Rifat Atun

**Affiliations:** 1 NOVA Center for Global Health NOVA Information Management School Universidade Nova de Lisboa Lisbon Portugal; 2 Department of International Health, Care and Public Health Research Institute Faculty of Health, Medicine and Life Sciences Maastricht University Maastricht Netherlands; 3 Department of Health Policy and Management Harvard T.H. Chan School of Public Health Harvard University Boston, MA United States

**Keywords:** influenza, gamification, public health policies, vaccination coverage rates, health promotion

## Abstract

**Background:**

Influenza represents a critical public health challenge, disproportionately affecting at-risk populations, including older adults and those with chronic conditions, often compounded by socioeconomic factors. Innovative strategies, such as gamification, are essential for augmenting risk communication and community engagement efforts to address this threat.

**Objective:**

This study aims to introduce the “Let’s Control Flu” (LCF) tool, a gamified, interactive platform aimed at simulating the impact of various public health policies (PHPs) on influenza vaccination coverage rates and health outcomes. The tool aligns with the World Health Organization’s goal of achieving a 75% influenza vaccination rate by 2030, facilitating strategic decision-making to enhance vaccination uptake.

**Methods:**

The LCF tool integrates a selection of 13 PHPs from an initial set proposed in another study, targeting specific population groups to evaluate 7 key health outcomes. A prioritization mechanism accounts for societal resistance and the synergistic effects of PHPs, projecting the potential policy impacts from 2022 to 2031. This methodology enables users to assess how PHPs could influence public health strategies within distinct target groups.

**Results:**

The LCF project began in February 2021 and is scheduled to end in December 2024. The model creation phase and its application to the pilot country, Sweden, took place between May 2021 and May 2023, with subsequent application to other European countries. The pilot phase demonstrated the tool’s potential, indicating a promising increase in the national influenza vaccination coverage rate, with uniform improvements across all targeted demographic groups. These initial findings highlight the tool’s capacity to model the effects of PHPs on improving vaccination rates and mitigating the health impact of influenza.

**Conclusions:**

By incorporating gamification into the analysis of PHPs, the LCF tool offers an innovative and accessible approach to supporting health decision makers and patient advocacy groups. It enhances the comprehension of policy impacts, promoting more effective influenza prevention and control strategies. This paper underscores the critical need for adaptable and engaging tools in PHP planning and implementation.

**International Registered Report Identifier (IRRID):**

RR1-10.2196/55613

## Introduction

### Influenza Epidemiology and Public Health Impact

Influenza is an infectious respiratory disease caused by an airborne virus. There are 4 identified types of influenza viruses, with types A and B being responsible for most of the seasonal influenza epidemics that occur annually [1,2]. The influenza virus can cause mild to severe disease, with risk groups (older people, pregnant women, young children, and individuals with chronic health conditions and autoimmune diseases) being more susceptible to severe forms of the disease [1].

The World Health Organization (WHO) estimates that annual influenza epidemics result in approximately 1 billion infections, 3 to 5 million cases of severe illness, and between 290 and 650,000 deaths [3]. The severity of influenza depends on multiple factors, including the virulence of the virus strain and the level of preexisting immunity in the population [1]. Influenza is also responsible for the worsening of previous health conditions in the individual and has consequences on different domains of individual health [4], such as cardiovascular [5], neurological, renal, respiratory, and diabetic complications.

However, despite the large number of respiratory infections worldwide, it is difficult to estimate the proportion of hospitalizations attributable to influenza across countries or over time [6]. During influenza outbreaks, health systems experience increased demand for services due to an influx of patients seeking medical care. This surge in patient demand can strain health care resources, including hospital beds, medical staff, and supplies [7]. As a result, the pressure on health systems during influenza outbreaks has an impact on access to care for other patients who require medical attention for non–influenza-related conditions, especially those at risk for complications [8].

Another study, published in 2023, which carried out a meta-analysis to improve understanding of the estimates of hospitalizations associated with influenza, concluded that seasonal influenza epidemics result in 3.2 million hospitalizations per year globally [9]. A study that evaluated 10 influenza seasons (between 2008 and 2018) in Portugal to estimate the clinical and economic costs of influenza reported that, on average, hospitalizations due to influenza were 11.6 cases per 100,000 inhabitants [10].

### Vaccination for Influenza Prevention

Vaccination against influenza began in the early 1940s, marking a considerable advancement in public health with the introduction of trivalent vaccines [11]. This approach evolved, and in 2012, quadrivalent vaccines were introduced [11], offering broader protection against the influenza virus. The WHO is currently deliberating on the possibility of returning to trivalent vaccines [12], showcasing the ongoing evolution and reassessment of strategies to combat influenza. The effectiveness of the influenza vaccine, which varies between 40% and 60% in the general population, depends on various factors, including patient characteristics such as age and underlying health conditions, as well as the match between the circulating influenza viruses and the seasonal influenza vaccination administered [13].

Recognizing influenza as a considerable global public health challenge, the WHO has advocated for annual influenza vaccination as the most effective measure to combat the influenza. Priority is notably given to older population groups, who face an increased risk of severe influenza disease. However, the effectiveness of influenza vaccinations in these groups is compromised by immune senescence, leading to diminished antibody responses. In response, higher-dose influenza antigen vaccines have been developed, enhancing the immune response and proving to be more effective in preventing influenza infections [14], hospitalizations, and reducing mortality rates [15] compared with standard-dose vaccines.

This targeted approach to vaccination aligns with the WHO’s Global Influenza Strategy 2019 to 2030 [16], which recommends a goal of increasing vaccination coverage rates (VCRs) for influenza to 75% by 2030, a goal also echoed by the European Union (EU) in a 2009 Council Recommendation [17]. This 75% target applies especially to older adults, extending as well to other high-risk groups, including persons with chronic conditions and health workers, advocating for comprehensive coverage to mitigate the impact of influenza. Despite concerted efforts, reaching this target has been challenging; as of 2018, influenza vaccination coverage among people aged ≥65 years was <50% in most EU countries, averaging only 39% across these nations and indicating that no country had yet met the 75% coverage target [18].

VCRs are crucially impacted by social inequalities, including financial, cultural, and linguistic barriers [19,20]. Evidence from a broad range of studies highlights this correlation, pointing out a concerning lack of data on VCRs among specific susceptible groups in various countries. The situation is further complicated by the interaction between multimorbidity and social exclusion, creating a vicious cycle where social inequality exacerbates health disparities, thereby deepening social vulnerabilities [21-23]. This interconnection between social determinants and health outcomes underscores the necessity of addressing these challenges holistically to enhance VCRs and strengthen the global response to influenza.

In this context, health management strategies implemented by health and governance authorities, alongside other health decision makers, are pivotal. Public health policies (PHPs) play a key role in managing potential risks by preempting and mitigating their impacts. Vaccination-related PHPs exemplify this approach effectively; by fostering immunity within the community, they not only reduce the likelihood of disease transmission but also ensure that any infections are less severe. This diminishes the demand for hospitalization and decreases mortality risk. Such policies, therefore, are not merely preventive measures but also crucial interventions that address the root causes of health inequities, aiming to break the cycle of multimorbidity and social exclusion by ensuring equitable access to vaccination and health care services.

### Gamification Strategies in Public Health

In the realm of scenario planning and policy development, traditional methodologies have steadily evolved to incorporate advanced technological capabilities, facilitating a more integrated and dynamic approach to learning and assessment. The advent of gamification, defined as the integration of game design elements in nongaming contexts to bolster engagement [24], represents a substantial leap forward in this evolution.

At its core, gamification uses game design elements in nongaming scenarios to augment engagement levels. As delineated in the study by Brangier and Marache-Francisco [25], the gamification design process is anchored in the principles of human-computer interaction, underscoring the importance of user-centric design and interaction. This approach to gamification has gained traction across various sectors, including education, health care, marketing, and human resources, evidencing its versatility and widespread applicability.

However, a substantial fraction of gamification initiatives stumble, primarily due to flawed design strategies. This underscores the criticality of a well-articulated design process, attuned to the nuances of user engagement and interaction dynamics. Moreover, the aspect of measurement emerges as a pivotal consideration in gamification, advocating for a dynamic approach toward redesigning gamified experiences through real-time feedback and user data analytics. However, the adoption of metrics as a quantification tool remains inconsistently applied across gamification frameworks, highlighting an area ripe for further exploration and standardization [24].

The application of gamification in educational contexts, particularly for fostering sustainable behaviors, stands out as a promising approach. Despite existing knowledge gaps, empirical evidence attests to the efficacy of gamification in elevating motivation, engagement, and satisfaction among learners. This enhanced learning environment, in turn, cultivates a fertile ground for the dissemination of sustainability principles and other educational content, demonstrating the transformative potential of gamification as a methodological tool in learning scenarios [26].

These advancements have rendered the simultaneous quantitative and qualitative modeling of policies affecting various facets of the health system and wider society not only feasible but also widely accessible. This confluence of technology and methodology paves the way for an enriched understanding and assessment of policies through sophisticated simulations that blend numerical data with narrative contexts, thereby facilitating a holistic view of potential outcomes and impacts.

Despite this growing interest in the integration of game-like elements into learning scenarios, especially within the health sector, focused design frameworks for health education through gamification remain underdeveloped. This area presents a unique opportunity for pioneering health-specific frameworks that emphasize prototyping, experimentation, measurement, and continuous iteration. Such frameworks are crucial for refining gamification strategies to ensure their effectiveness and relevance in the use of gamification in the health sector [27].

Adopting gamification in PHP decision-making introduces a complex landscape of ethical implications that warrant careful consideration. Despite its potential to enhance public health initiatives by promoting behavior change and increasing engagement being considerable, it also raises concerns regarding data privacy, and informed consent.

Data privacy emerges as a critical concern, as gamification strategies often rely on collecting and analyzing personal health information to personalize interventions and track progress. The ethical management of this data is paramount. According to the study by Mittelstadt et al [28], ensuring data protection and privacy in digital health interventions requires robust encryption methods and transparent data handling policies. Without stringent safeguards, there is a risk of unauthorized access to sensitive information, potentially leading to misuse.

Informed consent is another cornerstone of ethical considerations in gamification. Participants must be fully aware of how their data will be used, the nature of the gamified intervention, and its potential risks and benefits. As Thaler and Sunstein [29] argue in “*Nudge*,” while gamification can guide behavior in beneficial ways, it must not manipulate or coerce participants. Ensuring that consent is informed and voluntary preserves autonomy and respects individual decision-making.

Gamification can oversimplify complex policy issues if interpreted verbatim without critical thinking. Especially if used as a game of health policy–related challenges and not a way of rehearsing ideas, there is a risk of reducing nuanced issues to overly simplistic solutions. This oversimplification can lead to misinterpretations and misapplications of policy measures, potentially undermining the effectiveness of interventions and disregarding the intricacies of health outcomes. In addition, given that gamification strategies often use rewards, incentives, and competition to drive behavior, its use in health-related contexts may impact individuals’ autonomy and decision-making if it is not framed in critical thinking. Therefore, in the development of this tool, under no circumstances does the individual receive any form of retribution for use or performance.

### The “Let’s Control Flu” Tool

In the intricate balance between ethical considerations and the exploratory potential of gamification, the “Let’s Control Flu” (LCF) tool emerges as a digital, interactive tool designed to support policy decision-making specific to influenza. The LCF tool aims to help enhance VCRs and fulfill the objectives outlined in the WHO’s Global Influenza Strategy 2019 to 2030. Rooted in the qualitative framework suggested in the study by Kassianos et al [30], this project seeks to align VCR achievements with the benchmarks established by the WHO and the Council of Europe. The LCF tool demystifies the creation of epidemiological scenarios, eliminating the need for users to possess in-depth knowledge of epidemiological modeling techniques or to engage with complex data sets, such as extensive statistical series. This user-friendly approach facilitates a more accessible and informed decision-making process for policy makers striving to combat influenza through increased vaccination uptake.

The LCF tool innovatively applies gamification principles to assist stakeholders in understanding the nuances of different PHPs regarding influenza vaccination. It achieves this by providing a simulated environment where users can experiment with various policy scenarios, thus offering insights into the potential impact on VCRs before their implementation. This simulation enables stakeholders to grasp the intricacies, trade-offs, and consequences of their policy decisions, fostering a deeper understanding and facilitating more informed choices about the PHPs to be adopted and executed. The engaging and interactive presentation of information within the LCF tool simplifies the comprehension of policy impacts, encouraging decisions backed by data-driven insights.

The limitations sector of this paper acknowledges the constraints and challenges faced by the LCF tool. Despite its innovative approach and initial success, the tool’s effectiveness and applicability are subject to ongoing evaluation and refinement. Originally piloted in Sweden, the LCF tool’s application has proven successful, marking an important first step in its practical use. Following this initial implementation, efforts are underway to broaden the tool’s application across 3 additional European countries. This expansion is aimed at enhancing the tool’s robustness and increasing its relevance across different geographic contexts, thereby contributing valuable insights to the global effort to improve influenza vaccination coverage through informed policy making.

## Methods

### Overview

The development of the tool was underpinned by an integrated epidemiological model, encapsulating the burden of influenza disease alongside the predictive impacts of selected PHPs on targeted populations. Initial steps in tool development involved a critical reduction in the number of policies under consideration: on the basis of the foundational work by Kassianos et al [30], the scope was narrowed from 42 to 13 policies, spanning across the 5 pillars identified in their research (Multimedia Appendix 1). This refinement was necessitated by the current state of knowledge, which does not allow for a discrete modeling of the effects attributable to each of the 42 PHPs initially proposed. Consequently, a strategy was used to merge closely related PHPs into coherent bundles, maintaining the original pillar-based categorization to ensure methodological orthogonality.

**Figure 1:**
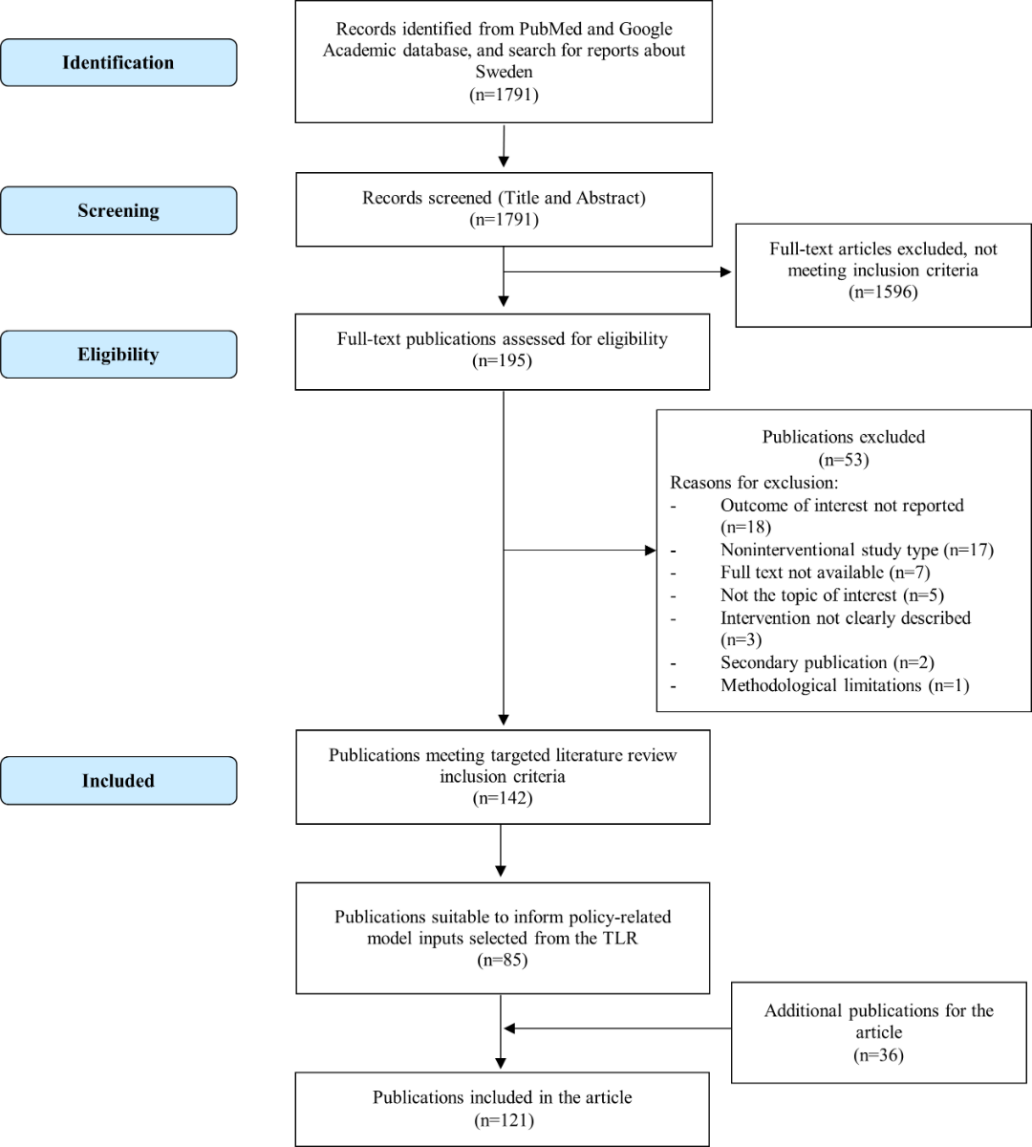
PRISMA (Preferred Reporting Items for Systematic Reviews and Meta-Analyses) flowchart of systematic literature review process. TLR: targeted literature review.

To ascertain the robustness and reliability of the model, we used a rigorous selection criterion for the scientific literature included in our analysis. Detailed in Textboxes 1 and 2, the inclusion and exclusion criteria were designed to encompass only the most pertinent and high-quality studies. In adherence to the PRISMA (Preferred Reporting Items for Systematic Reviews and Meta-Analyses) guidelines, we executed a systematic and comprehensive literature review. The PRISMA flowchart (Figure 1) delineates our methodical approach to the selection of publications, underscoring the scrutiny at each phase of article identification, screening, and inclusion. It is pertinent to note the exclusion of data from 2021 and 2022 to mitigate the confounding effects of the COVID-19 pandemic on the influenza landscape [31].

**Inclusion criteria for scientific works included in the model.**

**Inclusion criteria**

- Only pre-post intervention or controlled studies were included, enabling the effects of the policy to be effectively assessed independently of any background (and potentially not described) policies already in place.
- Studies were included whenever they contained data on one or more of the populations of interest for the model (children, health professionals; pregnant women; older adults, including adults aged ≥65 years and adults aged between 50 and 64 years; and high-risk patients).
- Studies reporting seasonal vaccination uptake were included, as opposed to pandemic influenza vaccination. This restriction was placed to ensure comparability of studies used to inform the model (ie, avoid pooling data for seasonal and pandemic influenza, as the coverage may not be comparable).

**Exclusion criteria for scientific works included in the model.**

**Exclusion criteria**

- Reviews were not included.
- Articles in any other language than English.

The interventions delineated in the included studies were meticulously aligned with the PHP categories identified in the study by Kassianos et al [30]. To ensure the validity and accuracy of the model construction, a National Advisory Board (NAB) comprising 4 eminent epidemiologists and public health experts from the Swedish health system was constituted. The board’s formation was predicated on their prior contributions to scientific literature and health authority reports over the last decade, ensuring their expertise was both relevant and current. The NAB was instrumental in the validation process, meticulously verifying the model-generated values and endorsing the results. This process was crucial, particularly in instances where the scientific literature did not provide ecologically valid data to substantiate the selection of proxies necessary for the tool’s development.

### Public Health Policies

The set of 13 PHPs incorporated into the LCF model maintained the original categorization into 5 pillars proposed by Kassianos et al [30], each targeting different aspects of public health to enhance vaccination rates and mitigate the health impacts of influenza.

#### Pillar 1: Health Authority Accountability and Strengthening the Influenza Program

VCRs targets set at national and regional levels for recommended population: To boost influenza vaccination rates [32], policies set targets at national and regional levels for specific population groups [33]. In Sweden, eg, this policy was implemented nationwide. Strategies programs [34-36] include educating individuals about vaccine benefits, improving vaccine accessibility, and offering incentives for vaccination, all aimed at increasing vaccine uptake.

Funding of influenza vaccinations for all recommended groups: To enhance influenza vaccine access, reduce the burden of disease, and prevent influenza-related deaths, immunization programs [37-41], intervention campaigns, and strategies [32,42-50] can be created. In addition, funds may be allocated to cover the cost of influenza shots for vulnerable groups, such as older adults, children, health care workers, and individuals with underlying health conditions. By doing so, public health officials improve vaccine accessibility and safeguard those with a higher risk of flu complications.

Nationwide regular monitoring of patient VCR at vaccination site and at health care professional (HCP) level by health authorities: Regular tracking of vaccine uptake in designated health facilities such as clinics, hospitals, and pharmacies [51,52] allows for monitoring immunization program effectiveness and identifying areas of improvement. By monitoring immunization coverage rates, public health authorities can use data to enhance programs and achieve desired health outcomes for the population.

HCP VCR as part of performance criteria in hospitals: Boosting vaccine uptake among health care workers and minimizing disease transmission through vaccination. This policy mandates that health worker immunization coverage rates be used as a performance criterion in health care facilities, including hospitals and primary care settings [53]. It encourages health care workers to get vaccinated, prioritizing their well-being and minimizing disease spread among patients, thereby fostering a safer and healthier environment for all.

Sustainable procurement system to ensure appropriate vaccine supply: Ensuring consistent and reliable availability of necessary vaccines for all individuals in need [54], irrespective of their location or financial circumstances. This policy aims to establish a procurement system that effectively manages vaccine acquisition, storage, and distribution to health units, considering cost, quality, and sustainability factors.

#### Pillar 2: Facilitated Access to Vaccination

Access to multiple vaccination settings: Enhancing vaccine access and reducing barriers to vaccination ensures that individuals can receive vaccines in various locations, including hospitals [48,53,55-57], clinics [36,58], pharmacies [39,59-62], schools [43,52,63-70], and other settings [46,71]. By doing this, public health authorities increase convenience and improve the chances of individuals getting vaccinated.

Call-to-action communications to target groups by multiple stakeholders: This policy targets influenza-related stakeholders, including public health agencies, health professionals, schools, and community organizations. Various methods such as email [69,70,72-74], SMS text messages [75-81], phone calls, health apps, and others [49,82] are used to remind target groups about the importance of vaccination. The objective is to boost vaccine uptake by reminding individuals in target groups of vaccination’s significance and encouraging them to get vaccinated [83-86].

HCP pop-up notification or SMS text message to population to vaccinate eligible patients: Health care providers use pop-up notifications or SMS text messages to remind eligible patients of the importance of vaccination and encourage them to get vaccinated [84,85,87-89], thus increasing vaccine uptake.

#### Pillar 3: HCP Accountability and Engagement

Regular HCP education and training: This policy ensures HCPs, including doctors, nurses, and other professionals, receive ongoing education and updates on new technologies, medical advancements, and changes in vaccination practices [33,34,48,65,77,87-94]. Its objective is to equip HCPs with the necessary knowledge and skills to deliver safe, effective, and high-quality care to patients.

Fair and specific HCP compensation per vaccination: Establishing a system where health professionals receive fair compensation for administering vaccines [95]. This aims to incentivize HCPs to administer vaccines and ensure they are adequately rewarded for their time and efforts. Fair compensation not only supports HCPs but also encourages their dedication to protecting public health.

Mandatory HCP vaccination: Mandating or strongly recommending that health care workers receive specific vaccines as part of their job [35,36,53,96-106]. This policy aims to safeguard public health by minimizing the transmission of communicable diseases in health care settings such as clinics or hospitals, particularly to patients with weakened immune systems. By requiring or strongly recommending vaccination for HCPs, the policy aims to prevent them from being carriers of diseases that could be transmitted to their patients.

#### Pillar 4: Awareness of the Burden and Severity of the Disease

Coordinated multistakeholder awareness and communication campaigns: This policy promotes immunization through collaboration and coordination among various stakeholders, including government agencies, hospitals and other health organizations [33,34,46,47,52,57,68-71,77,83,86-89,91,97,105, 107-110], health professionals [32,41,42,58,65,72,111,112], community organizations, schools, and universities [43,113], as well as the media [105,114]. Its goal is to deliver a cohesive message about the importance of immunization, reaching a wide audience with information on the benefits of immunization and how to access it. By addressing concerns, dispelling misinformation, and creating a supportive environment [115,116], the policy aims to enhance vaccine uptake.

#### Pillar 5: Belief in Influenza Vaccination Benefits

Positive media coverage of vaccines: The policy promotes a positive approach to vaccine promotion, highlighting the benefits of vaccination and countering negative or misleading information. It aims to foster a vaccine-friendly environment by showcasing the safety, efficacy, and importance of vaccines through various media channels, such as news articles, public service announcements, and other media content [41]. The policy’s purpose is to boost vaccine uptake by offering accurate information, debunking myths, and empowering the public to make informed decisions about vaccination.

### Model Design

The model categorizes the population into 6 age groups—12 to 14, 15 to 34, 35 to 49, 50 to 64, and >65 years—and identifies 5 key target groups: children (aged 0-14 years), older adults (aged >65 years), health professionals, pregnant women, and high-risk patients (aged 15-64 years with ≥1 chronic condition). This segmentation acknowledges the critical challenge of chronic undervaccination in high-risk patients, which poses substantial barriers to achieving optimal health outcomes and protection against influenza and its complications [117].

To assess the impact of PHPs on these target groups, the model simulates 7 health outcomes over a 10-year horizon (2022-2031), including influenza infections averted, hospitalizations averted, influenza-related general practitioner visits averted, workdays lost (productivity impact), influenza-related deaths averted, hospitalizations averted due to cardiovascular complications, and deaths averted due to cardiovascular complications. These outcomes are evaluated both across the total population and within each target group, providing a comprehensive overview of PHP effectiveness in mitigating influenza-related morbidity and mortality.

In the development of the epidemiological model, weights were assigned to each policy across different target groups and specified time frames using a multifaceted approach. This methodology enabled a rigorous, evidence-based evaluation of PHPs’ impact on VCRs, accounting for the variability and complexity of policy effects across different demographic and risk groups. It encompassed the following:

1. Policy effects derived from the literature: weights were calculated as weighted averages for each policy and target group based on a comprehensive review of the existing literature. This approach ensures that the model’s parameters are grounded in empirical evidence, reflecting the documented effects of PHPs on VCRs.
2. Imputation from similar policies or different target groups: in instances of incomplete data for a specific policy within a target group, weights were imputed based on the relative effects of the most analogous policies available. Alternatively, effects documented for the same policy in different target groups were adjusted and applied, facilitating a coherent extrapolation of policy impacts.
3. Projected increases for subsequent years: for policy effects lacking data beyond the first year, a conservative projection was applied, assuming a 10% increase in the second year and a 20% increase for the third year onward. This assumption is based on the initial year’s effect, providing a structured approach to model the temporal dynamics of policy impacts.
4. Integration of expert opinions: insights and recommendations were solicited from the NAB to inform the weighting process, particularly in areas where empirical data were sparse or ambiguous. This inclusion of expert judgment ensures that the model remains adaptable and relevant to current public health contexts, incorporating the nuanced understanding of seasoned professionals in the field.

When policy effects were extrapolated from the literature, the inherent challenges associated with integrating qualitative findings directly into quantitative models led to the necessity of generating temporary proxies to bridge the gap between qualitative insights and the quantitative demands of the model. This process was initiated with comprehensive discussions within the scientific coordination team of the project, after which the proposed proxies underwent a rigorous phase of deliberation and validation by the NAB, ensuring that the proxies accurately reflect the intended policy effects. The final step in this process involved the validation of the proxies by the project’s external scientific adviser, who evaluated their suitability and alignment with the model’s objectives and the underlying empirical evidence. This layered approach to proxy generation and validation ensured the model’s integrity and the reliability of its projections, accommodating instances where direct quantitative data had been lacking.

In scenarios where explicit data concerning the uncertainty of parameters was unavailable, our approach entailed the adjustment of these parameters through the application of suitable statistical distributions, premised on an SE estimated at 10% of the base-case value. This procedure was designed to embed a quantifiable measure of uncertainty into the model, thereby ensuring a broader and more realistic spectrum of potential outcomes.

The model was also designed with a flexible, modular structure, making it easy to update with new scientific findings without having to change its core algorithm. This flexibility is crucial for continually improving the model, allowing regular updates to its parameters and data. The modular design also supports replacing preliminary estimates with actual, up-to-date information, which improves the model’s accuracy and relevance as new research becomes available.

**Figure 2:**
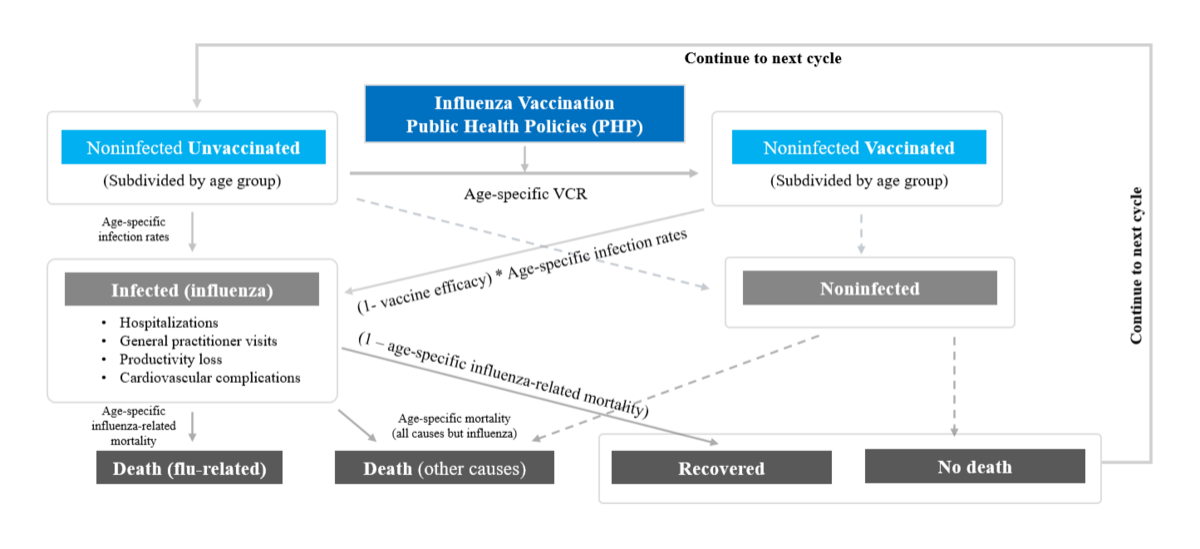
Graphic representation of the model adopted to estimate the effect of the 13 elected public health policies. VCR: vaccination coverage rate.

Figure 2 showcases the compartmental model designed to elucidate the dynamics among vaccination strategies, influenza transmission, and consequent health outcomes. This model delineates the population into distinct compartments, primarily focusing on the age-stratified cohort of noninfected individuals who are initially unvaccinated. Upon the application of vaccination policies, a transition of these individuals into the vaccinated cohort is anticipated, contingent upon their noninfection status, which predicates the absence of health complications or influenza-induced mortality, thereby facilitating a cyclic annual assessment. In the event of influenza infection, the model accounts for resultant health implications, including hospital admissions, general practitioner consultations, loss in work productivity, and cardiovascular complications, using a formulaic approach that integrates vaccine efficacy and age-specific infection rates. Identical principles extend to the unvaccinated population who contract the virus, after which the model delineates 3 potential pathways postinfection: mortality attributable to influenza, mortality from alternate causes, or recovery. The recovery probabilities are quantified through a formula that encapsulates influenza-associated mortality rates across different age groups.

### The Operational Functionality of the Tool

The design of this tool prioritized accessibility and user-friendliness, targeting a wide audience that spans HCPs, the public, and patient advocacy groups. By facilitating an interactive platform, it enables users to easily simulate the impact of selecting from the 13 distinct PHPs on various health outcomes within specific target groups, for any year between 2022 and 2031.

Upon the selection of policies, a prioritization mechanism is applied where the weight of each subsequent policy decreases fractionally to reflect its comparative impact on the overall scenario. This methodology underscores the varying significance of individual policies and their synergistic effects when combined. This system also incorporates a realistic element of societal resistance to change, adjusting the cumulative weight of selected PHPs to reflect this factor.

Furthermore, the tool bases its analysis on existing PHPs within a country, recognizing prior efforts and their varying degrees of success. The simulation outcomes represent the scenario of maximum policy efficacy, acknowledging that complete population compliance is an ideal rather than a constant reality due to people’s inherent resistance to change. The possibility of achieving perfect policy implementation is speculated for future contexts, suggesting that even in such ideal conditions, there is potential for further enhancement. This could be achieved through the adoption of new communication and educational strategies leveraging emerging technologies, adapting to changing societal contexts, and more effectively engaging target populations.

### Ethical Considerations

The LCF project was initiated at the Catholic University of Portugal; was subject to the code of ethics NR/R/1419/2015 at the time; and was later transferred, together with the team, to the NOVA-IMS Faculty of the Universidade Nova de Lisboa, where it was subject to the supervision of its ethics committee, in accordance with the Universidade Nova de Lisboa code of ethics (approval 15464/2014) published on December 19, 2014. Access to data was only granted to published and public material, and there was no access to personal data or any other data that could be used to identify someone.

### Democratizing Access and Combating Misinformation

The development of this tool democratizes access to complex epidemiological scenario modeling, allowing users without advanced technical knowledge of PHPs and health impact modeling to explore the potential effects of different health policy scenarios. This capability not only streamlines the decision-making process for health policy but also upholds the principle of technological transparency, ensuring that the underlying mechanisms and assumptions of the tool are clear and understandable.

The dedicated website [118] hosting the LCF tool serves as an educational resource, offering scientifically accurate information about influenza and vaccination in accessible terms. This approach is instrumental in combating misinformation and reducing vaccine hesitancy, thereby supporting global efforts to lessen the impact of seasonal and pandemic influenza [119]. In this context, it is also relevant to acknowledge the differences between vaccine resistance and vaccine hesitancy [120,121], where vaccine resistance often stems from deeply rooted ideological, religious, or philosophical beliefs and, in contrast, vaccine hesitancy is typically driven by fear and doubts, frequently fueled by misinformation or distorted news. Recognizing these differences is crucial for tailoring intervention strategies effectively, as in contexts where vaccine resistance is prevalent due to entrenched beliefs, efforts may need to focus on long-term engagement and education; conversely, in areas where hesitancy predominates, addressing misinformation and building trust through transparent communication may prove more effective. Understanding these nuances is essential for optimizing the impact of our approach across diverse populations, as applying this tool in different legal and cultural contexts may face increased inertia from the public.

## Results

The LCF project, initiated in February 2021, is projected to conclude in December 2024. The phase focused on model creation and its implementation in the pilot country, Sweden, occurred from May 2021 to May 2023.

The pilot phase of the project was concluded successfully and revealed positive outcomes on the Swedish population. For example, the use of the tool to simulate the comprehensive application of all 13 PHPs across various target populations for the year 2025 provided insightful projections, as the results demonstrate a substantial potential increase in the national influenza VCR, from 18.6% (1,940,964/1,042,283,400) to 28.4% (2,962,903/1,042,294,300). This comprehensive approach revealed increases in VCRs across all specified target populations: older adults experienced a substantial increase to 75% (1,636,707/218,227,600) increase, as well as in high-risk patients who also achieved 75% (825,076/110,010,100), pregnant women’s VCR went up to 57.3% (61,894/10,804,700), and health workers saw an increase to 53.1% (276,466/52,085,500). Children VCR went up to 0.8% (15,907/179,057,900) rise, representing an increase of 50% from the baseline value, knowing that high-risk children are vaccinated. Moreover, the anticipated health benefits from this full-scale policy implementation are considerable, including the avoidance of 21,935 influenza infections and 277 hospitalizations. In addition, there would be 5871 fewer physician visits, a reduction of 19,829 workdays lost due to influenza, 38 lives saved from influenza-related deaths, 62 hospitalizations prevented due to cardiovascular complications, and ultimately, 8 fewer deaths from cardiovascular issues related to influenza.

## Discussion

### Principal Findings

Gamification is an innovative approach that can play a considerable role in improving PHP decision-making. By using gaming mechanics and design, gamification can make complex information and issues more accessible and engaging, leading to a greater understanding of public health challenges, namely for health policy makers who come from outside the professional health sphere and need support tools for their decision-making that provide them with a decision that is best supported by science. Gamification can also create opportunities for active participation and collaboration, allowing individuals to actively shape PHPs and decisions. In addition, gamification can facilitate the collection of real-time data and feedback, providing valuable insights into the impact of PHPs and informing decision-making processes.

By combining play with the need for informed policy decisions, gamification has the potential to positively impact public health and help ensure that policies are both effective and evidence based and also helps to address social determinants of health by engaging disadvantaged communities and increasing their access to health information and resources. Through interactive experiences, gamification can help to break down barriers and increase health literacy, which can lead to better health outcomes. Furthermore, gamification can foster a sense of community and collaboration among individuals, which can help to build a shared understanding of the importance of public health and create a culture of health.

### Limitations

In this paper, we delineate several principal limitations inherent to the use of gamification strategies within the realm of predictive modeling. First, we must acknowledge the ethical and methodological implications posed by gamification: these methodologies do not offer precise forecasts of future events but rather serve as heuristic guides. They project potential futures predicated on historical data, presuming all other variables remain constant (ceteris paribus). This deterministic nature inherently restricts the model’s ability to predict future impacts with absolute certainty.

Second, the foundation of such modeling lies in data analysis, which encounters substantial challenges regarding the availability and quality of data. This is particularly pronounced at the local level, where the procurement and qualification of data sources, often designed for disparate purposes within complex ecosystems, necessitate the generation of proxy measures. Despite the rigorous and meticulous process of producing and validating these proxies, they introduce inherent risks and uncertainties into the modeling efforts.

Third, we must consider the impact of abrupt contextual shifts that can fundamentally disrupt the health care management landscape, thereby breaching the fundamental principles of modeling. The COVID-19 pandemic serves as a striking example, illustrating how populations initially compliant with national vaccination strategies may suddenly exhibit resistance due to widespread disinformation campaigns. This phenomenon has led to a decline in VCRs for numerous vaccines, culminating in the resurgence of diseases such as polio in locations such as London and New York, a scenario deemed inconceivable a decade ago.

In addition, our model does not account for the influence of other policies that may directly or indirectly affect health outcomes. These include frameworks for occupational health, such as specific recommendations and obligations within various work contexts, as well as the management of health issues within institutions focused on older adult care. Furthermore, the model does not incorporate the integration of health issues into broader economic and policy areas, commonly referred to as Health in All Policies. Despite being endorsed by the EU since 2011, Health in All Policies has seen limited practical implementation. Such policy integration could have a considerable impact on vaccination rates, not only for influenza but also for other vaccines administered throughout an individual’s life. Consequently, our model does not capture this dynamic aspect of health management that extends beyond the specific scope of influenza vaccination.

Given these limitations, we advocate for the periodic reassessment of the modeling framework, recommending an annual (or, ideally, biannual) review. This process is intended to incorporate the latest bibliographic resources and replace proxy measures with evidence-based values. Such a practice not only ensures the model’s ongoing refinement but also contributes to the accumulation of evidence necessary for evaluating health impacts in the context of vaccination policy development or modification.

### Conclusions

The field of work on increasing VCRs lends itself well to gamification because (1) general trust in vaccines has been deeply shaken by misinformation and fake news during the COVID-19 pandemic, and it is possible to demonstrate the immense advantages that good vaccine coverage provides; (2) in particular, influenza is one of the diseases with the highest pandemic potential, and only through vaccination against the disease will it be possible to mitigate its potential effects; and (3) the entire community, from policy makers, often disconnected from health issues, to the most susceptible citizen, can understand how and where the benefits of influenza vaccination work.

The journey toward improving VCRs globally is not solely a medical challenge but a multifaceted endeavor that requires addressing the underlying social determinants of health. The intertwined relationship between social inequalities and multimorbidity highlights a critical area for intervention. As evidenced, these disparities in social, financial, cultural, and linguistic domains not only hinder equitable access to vaccination but also exacerbate the vulnerability of marginalized communities, thereby impeding the overall effectiveness of influenza vaccination efforts. Therefore, it is imperative that strategies to enhance VCRs also focus on increasing literacy, especially in health-related matters. Empowering individuals with knowledge and understanding of the importance of vaccination can drive more informed decisions, leading to higher uptake rates. In addition, concerted efforts to combat social inequalities are paramount. By creating more inclusive health systems and policies that actively address the barriers faced by disadvantaged groups, a way can be paved for a more equitable distribution of health resources, including vaccines. Ultimately, enhancing health literacy and addressing social inequalities stand as potential catalysts for boosting VCRs, thereby strengthening the global response to influenza and enhancing public health outcomes for all communities.

The design and implementation of the LCF tool have shown promising results in facilitating a broad and user-friendly approach to evaluating PHPs impacts on influenza VCRs and health outcomes. Its capacity to simulate the effects of various PHPs, considering both individual significance and collective synergy, highlights the tool’s utility in strategic health planning. Furthermore, the tool’s acknowledgment of societal resistance and its foundation on existing PHPs underscore the complexity of health policy efficacy. By offering a platform for dynamic scenario testing, the tool not only assesses current strategies but also identifies opportunities for enhancement, reinforcing the importance of continual innovation and adaptability in public health initiatives. This approach underscores the evolving nature of health strategies and the critical need for responsive, technology-driven solutions to meet public health challenges effectively.

In our interpretation, we see potential for developing the tool in several directions: (1) through its application in other legal and cultural contexts; (2) giving it new capabilities, namely the possibility of cost assessment; (3) adapting the tool to other human vaccination contexts, namely those with low or very low VCRs, as is generally the case with adult vaccination; and (4) developing the gamification capacity used in this context for other public health situations, namely in the prevention of chronic diseases.

## Appendix

Multimedia Appendix 1 Public health policies of the “Let’s Control Flu” model, organized by Pillar, based on the study by Kassianos et al.

## Abbreviations

EU: European Union
HCP: health care professional
LCF: Let’s Control Flu
NAB: National Advisory Board
PHP: public health policy
PRISMA: Preferred Reporting Items for Systematic Reviews and Meta-Analyses
VCR: vaccination coverage rate
WHO: World Health Organization
