# Supplementary material for: Digital Gamification Tool (Let’s Control Flu) to Increase Vaccination Coverage Rates: Proposal for Algorithm Development
Source: JMIR Res Protoc. 2024 Sep 10;13:e55613. doi: 10.2196/55613 (PMC11422745; doi:10.2196/55613)
Supplement: Multimedia Appendix 1 [file resprot_v13i1e55613_app1.docx]

| **Pillar 1:**  **Health authority accountability and strengths of the influenza immunisation programme** | **Pillar 2:**  **Facilitated access to vaccination** | **Pillar 3:**  **HCP accountability and engagement** | **Pillar 4:**  **Awareness of influenza burden and severity of disease** | **Pillar 5:**  **Belief in influenza vaccination benefits** |
| --- | --- | --- | --- | --- |
| HA leaders willing to champion influenza vaccination | Access to multiple vaccination settings | Regular HCP education and training by multiple stakeholders | Structured and robust influenza surveillance network | Overall trust in influenza vaccine safety and effectiveness |
| VCR targets set at national and regional levels for recommended populations | Multiple HCPs allowed to vaccinate target population | Fair and specific HCP compensation per vaccination | Reliable collection and dissemination of data on influenza burden | Trust in the influenza vaccine as the most effective prevention |
| Nationwide regular monitoring of patient VCR at vaccination site/ HCP level by HA | Convenient and seamless vaccination journey for all target populations | Attractive VCR-linked financial incentive for HCP | Proven evidence of the economic direct and indirect impacts of influenza | Public trust in HA and HCP communication |
| Data collection and reporting on HCP vaccination status | No financial barriers to getting immunised (i.e. no out-of-pocket expenses or cash layout) | Individual vaccination status visibility across providers (e.g. GP, pharmacist) | Published data on influenza-related disruption of the healthcare system and company productivity | Positive media coverage of vaccines |
| HCP VCR as part of performance criteria in hospitals and primary care | Awareness of vaccine recommendations by target populations | Competition through publication of VCR at vaccination area/HCP level | Coordinated multistakeholder communication campaigns | Effectively dealing with active anti- vax groups |
| Multistakeholder coalition supporting influenza immunisation | Reminder call-to-action communications to target groups by multiple stakeholders | Mandatory/strongly recommended HCP vaccination | Patient associations actively support influenza vaccination | Monitoring and responsiveness vaccine disinformation |
| Investment in pandemic preparedness | Vaccine dedicated refrigerators at vaccination setting (e.g. GP practice) | Simple influenza vaccine procurement process for GPs | Target populations motivated to get vaccinated |  |
| Systematic assessment of cost-efficiency of VCR initiatives | HCP pop-up notification to vaccinate eligible individuals | HCP associations actively endorsing influenza vaccination |  |  |
| Regional HAs willingness to develop new initiatives to drive VCR | Availability of influenza vaccines (including cold chain management) in close proximity to the patients | Clear guidance about vaccine-specific usage per target population |  |  |
| Sustainable procurement system to ensure appropriate vaccine supply |  |  |  |  |
| Funding of flu vaccinations for all recommended groups |  |  |  |  |

**Multimedia Appendix 1: Public Health Policies of the “Let’s Control Flu” Model, Organized by Pillar, Based on Kassianos et al. (2021).**

Kassianos G, Banerjee A, Baron-Papillon F, Hampson AW, McElhaney JE, McGeer A, et al. Key policy and programmatic factors to improve influenza vaccination rates based on the experience from four high-performing countries. Drugs Context. Jan 05, 2021;10:1-13. [FREE Full text] [doi: 10.7573/dic.2020-9-5] [Medline: 33456480]
